# Crystal structure of NucB, a biofilm-degrading endonuclease

**DOI:** 10.1093/nar/gkx1170

**Published:** 2017-11-20

**Authors:** Arnaud Baslé, Lorraine Hewitt, Alan Koh, Heather K Lamb, Paul Thompson, J Grant Burgess, Michael J Hall, Alastair R Hawkins, Heath Murray, Richard J Lewis

**Affiliations:** Institute for Cell and Molecular Biosciences, Faculty of Medical Sciences, Newcastle University, Newcastle upon Tyne NE2 4HH, UK; Centre for Bacterial Cell Biology, Faculty of Medical Sciences, Newcastle University, Newcastle upon Tyne NE2 4AX, UK; Marine Biology, School of Natural and Environmental Sciences, Newcastle University, Newcastle upon Tyne NE1 7RU, UK; Chemistry, School of Natural and Environmental Sciences, Newcastle University, Newcastle upon Tyne NE1 7RU, UK

## Abstract

Bacterial biofilms are a complex architecture of cells that grow on moist interfaces, and are held together by a molecular glue of extracellular proteins, sugars and nucleic acids. Biofilms are particularly problematic in human healthcare as they can coat medical implants and are thus a potential source of disease. The enzymatic dispersal of biofilms is increasingly being developed as a new strategy to treat this problem. Here, we have characterized NucB, a biofilm-dispersing nuclease from a marine strain of *Bacillus licheniformis*, and present its crystal structure together with the biochemistry and a mutational analysis required to confirm its active site. Taken together, these data support the categorization of NucB into a unique subfamily of the ββα metal-dependent non-specific endonucleases. Understanding the structure and function of NucB will facilitate its future development into an anti-biofilm therapeutic agent.

## INTRODUCTION

Free-living, motile bacteria can develop into a stationary, multicellular community of cells known as a biofilm, a colony of sessile cells that forms on natural or artificial moist surfaces (1,2). Medical implants and devices can be contaminated by biofilms (3), and dental caries (4), upper respiratory tract (5), ear infections (6) and chronic lung infections in cystic fibrosis patients are caused by biofilms (7,8). Soil-dwelling bacteria are associated with the biofilms of plants in both symbiotic and pathogenic relationships (9). Finally, biofilms are 10^1^–10^4^ times more resistant to antibiotics than free-living bacteria (10,11), and biofilms thus represent a significant world-wide challenge in society, healthcare, agriculture and industry.

A molecular glue, called the extracellular matrix (ECM), holds the biofilm together. The ECM is a relatively impermeable barrier of proteins, carbohydrates and extracellular DNA (eDNA) (1), and common themes and species-dependent differences in ECM formation in bacteria are now beginning to emerge (2). For instance, a subpopulation of *Bacillus subtilis* cells that are destined to sporulate (the ultimate survival mechanism for this species) are located at the periphery of the biofilm (2,12,13) akin to the fruiting bodies of fungi and myxobacteria. Degradation of the ECM results in biofilm dispersal. Nutrient levels, environmental factors and bacterially-derived small molecule effectors can all trigger signal transduction pathways that result in biofilm dispersal (14,15). Furthermore, secreted proteases (16) and glycoside hydrolases (17) degrade the protein and carbohydrate components of the ECM, respectively (15). eDNA is a critical component of the ECM (18–20) and is required for the initial adhesion phase (21). It was first shown several decades ago that biofilms treated with bovine DNase I had reduced viscosity (22) leading to biofilm dispersal (21). Indeed, cystic fibrosis can be treated with a nebulizer that contains recombinant human DNase I (Dornase Alfa) to reduce the viscosity of the patient's sputum to promote its clearance. There is increasing evidence that secreted nucleases play important roles in biofilm formation, dispersal and remodelling in many bacterial phyla, including major pathogens of humans such as *Pseudomonas aeruginosa* (7,8), *Vibrio cholera* (23) and *Staphylococcus aureus* (19).

An unidentified protein purified from *B. subtilis* cell lysates had been shown previously to degrade DNA in a divalent cation-dependent manner (24). This enzyme was found to be expressed in late stage II of sporulation (25) and was subsequently designated NucB (26). When a biofilm dispersing supernatant from a marine isolate of *Bacillus licheniformis* was analysed, one of the active compounds was found to be a NucB orthologue (*Bl*NucB) (27). This enzyme was able to disperse a broad range of mono- and mixed-species biofilms by degrading eDNA and may be useful in combatting a number of biofilm-related problems (3–5,27,28). However, NucB orthologues share no sequence homology to any other protein family and thus a molecular understanding of their biochemical properties is completely lacking. To shed light on the biofilm-dispersing properties of NucB, we present here an analysis of the mode of action of this novel nuclease, its crystal structure and a mutational analysis that confirms the active site. Together, these results classify NucB as the founding member of a novel subgroup of the non-specific His-Me finger endonuclease superfamily.

## MATERIALS AND METHODS

### Strains and plasmids

Unless otherwise stated all chemicals and reagents were obtained from Sigma-Aldrich. Nutrient agar (NA; Oxoid) and Luria-Bertani (LB) medium was used for routine selection and maintenance of *B. subtilis* and *Escherichia coli* strains (Supplementary Table S1). Standard techniques were used for strain construction (29). Transformation of competent *B. subtilis* cells with plasmid DNA (Supplementary Table S2) was performed using an optimized two-step starvation procedure as described previously (30,31). Transformation of chemically competent *E. coli* cells was performed as described (32). General manipulation of DNA was performed using standard procedures (33). To induce NucB expression, *B. subtilis* was grown in Schaeffer's medium (Nutrient broth (Difco), 1 mM MgSO_4_, 1 mg/ml KCl, 1 mM CaCl_2_, 130 μM MnSO_4_). Supplements were added as required: 20 μg/ml tryptophan, 5 μg/ml chloramphenicol, 2 μg/ml kanamycin, 10 μg/ml zeocin, 100 μg/ml ampicillin. Site-directed mutagenesis of *B. subtilis nucB* (*BsnucB*) was performed using primers listed in Supplementary Table S3. All plasmids and strains were verified by sequencing.

### Production and purification of *B. licheniformis* NucB

Recombinant *B. licheniformis* NucB (*Bl*NucB) proteins were prepared by expression in *B. subtilis* NZ8900 (27) and purified as described previously (4).

### Differential scanning calorimetry (DSC)

*Bl*NucB at a concentration of 34.3 μM in a buffer of 50 mM Tris–HCl (pH 8.0), 1 mM DTT was subjected to thermal unfolding in a MicroCal VP-DSC instrument. The scan range was 25–80°C, with a scan rate of 90°C/h. The raw data were deconvoluted using the non-two state model within MicroCal Origin (www.originlab.com).

### Crystallization, structure determination and refinement

Purified *Bl*NucB was concentrated by ultrafiltration to a concentration of 50 mg/ml for crystallization at 20°C by sitting-drop vapour diffusion using a Mosquito (TTP Labtech) liquid-handling robot and a series of commercially-available crystallization screens. The initial *Bl*NucB crystals, which were used for sulphur SAD phasing, crystallized in 200 mM sodium nitrate, 100 mM propionic acid/cacodylate/bis-tris propane buffer (pH 7.5), 20% (w/v) polyethylene glycol (PEG) 3350. *Bl*NucB crystals subsequently used for high resolution data collection were grown from 100 mM MES–NaOH (pH 6.5), 12% (w/v) PEG 20000. All samples were cryo-protected by supplementing the crystallization mother liquor with 20% PEG 400. The sulphur SAD diffraction data were collected on beamline I24 of the Diamond Light Source synchrotron at a wavelength of 1.907 Å to a maximum resolution of 2.26 Å; 999.9° from a single crystal rotated around φ were collected. Higher resolution data were collected on beamline I04 of the Diamond Light Source synchrotron at a wavelength of 0.9795 Å to a maximum resolution of 1.35 Å.

The data were integrated and scaled in XDS (34). Space group determination was confirmed with POINTLESS (35). The crystallographic phases for *Bl*NucB were determined by anomalous scattering from the sulphur atoms present in the protein chain with HKL2MAP (36) and the SHELXC/D/E suite (37), which correctly positioned four sulphurs, corresponding to the S atoms found in the two cysteines and two methionines in the sequence of mature *Bl*NucB. The structure was built automatically in ARP_wARP (38) and Buccaneer (39). Five percent of the observations were randomly selected for the *R*_free_ set, and model building and refinement cycles in Coot (40) and REFMAC5 (41) were interspersed until refinement reached convergence. The model was validated using Coot (40) and MolProbity (42). The data collection and model refinement statistics are summarized in Supplementary Table S4.

### Nuclease activity assays *ex vivo*

*B. subtilis* NucB (*Bs*NucB) proteins were natively expressed during sporulation. Cell cultures were inoculated into 2 ml of Schaeffer's media for 6 h at 37°C and these starter cultures were then diluted (1:100) into fresh Schaeffer's media and allowed to grow for 40 h at 37°C. Cells were pelleted and the culture supernatants were passed through a sterile 0.2 μm filter (Millipore) before analysis.

To determine the total nuclease activity present within each supernatant, *B. subtilis* genomic DNA (6 ng/μl; Qiagen DNeasy Kit) was combined with 10 μl of supernatants and incubated for 3 h at 37°C. The DNA samples were mixed with glycerol (5% final) and stained with 2X SYBR Gold (Thermo Fisher Scientific). The genomic DNA was separated using a 1% agarose gel run in TBE buffer (45 mM Tris–borate, 1 mM EDTA), and nucleic acid was visualized with a UV transilluminator.

### Nuclease activity assays *in vitro*

Purified *Bl*NucB was used to assess its nuclease activity *in vitro*. First, high molecular weight calf thymus (or salmon sperm) DNA was treated with *Bl*NucB; high molecular weight DNA is not soluble in 2% perchloric acid whereas products of the nuclease reaction <500 bp in length are acid-soluble, the absorption of which can be measured at a wavelength of 260 nm in an end-point assay (43). 5 ng of *Bl*NucB (final concentration 1.68 nM) was incubated at 37°C with 125 μg of calf thymus or salmon sperm DNA in a reaction buffer of 50 mM Tris–HCl (pH 8.0), 5 mM MnSO_4_ in a total reaction volume of 250 μl. The reaction was stopped after 60 min by mixing with an equal volume of ice-cold 4% (v/v) perchloric acid. The mixture was left to stand on ice for 40 min before the insoluble material (protein, high molecular weight DNA) was pelleted by centrifugation in a benchtop microfuge at 4°C. The supernatant was diluted 4-fold with 50 mM Tris–HCl (pH 8.0), 5 mM MnSO_4_ before the amount of low molecular weight DNA generated by *Bl*NucB was measured in a spectrophotometer at 260 nm using 1 cm pathlength quartz cuvettes. All reactions were performed in triplicate from single- to triple-preparations of proteins.

Second, supercoiled, relaxed and linearized pBR322 DNA were used as nuclease substrates. Linearized pBR322, exploiting the sole *Bam*HI restriction site in this plasmid, was generated by restriction using *Bam*HI (ThermoFisher) according to the manufacturer's instructions. pBR322 samples at final concentrations of 200 ng/μl were mixed with 0.33 ng of *Bl*NucB (final concentration 2.5 nM) in a reaction buffer of 10 mM Tris–HCl (pH 7.5), 5 mM MgCl_2_, 100 mM NaCl in a total reaction volume of 10 μl. Though it has been reported previously that maximal nuclease activity of the purified nuclease, presumed to be *Bs*NucB, was obtained in the presence of Mn^2+^ ions (24), we chose to use Mg^2+^ ions, dilute enzyme concentrations and to conduct the reactions at room temperature to potentially observe transient intermediates in the reaction trajectory. Samples were taken at time intervals and the nuclease reaction was stopped by the addition of EDTA to 10 mM and by heating to 95°C before the reaction products were separated by 0.8% agarose gel electrophoresis and visualized by GelRed (Biotium, USA) in-gel staining and UV transillumination.

Finally, *Bl*NucB was used against fluorescently labelled 30mer oligodeoxynucleotides (ATDbio, Southampton, UK) that incorporated two successive phosphorothioate linkages at either the 5΄-, 3΄- or both termini, with deoxythymidylyl-fluorescein isothiocyanate (dT-FITC) and/or deoxythymidylyl-tetramethyrhodamine (dT-TAMRA) incorporated at position 3 and/or 28, respectively. Stock solutions of single stranded oligodeoxynucleotides at 250 nM were prepared by re-solublizing the lyophilized DNA pellet in a hybridization buffer of 25 mM HEPES–KOH (pH 7.5), 100 mM KCl, 1 mM EDTA. Oligodeoxynucleotide stock solutions (100 μl) were mixed with an equal volume of the unlabelled complementary strand at 350 nM, heated to 85°C and then left to cool slowly overnight to yield double stranded oligodeoxynucleotides.

Single- and double-stranded fluorescent oligodeoxynucleotides were digested in a 25 μl reaction volume comprising 25 nM DNA and 100 ng of *Bl*NucB in a buffer of 10 mM Tris–HCl (pH 7.5), 5 mM MgCl_2_, 100 mM NaCl. At defined timepoints samples were taken and the reaction stopped with an equal volume of a quench solution at pH 7.5 comprising 95% formamide, 5% H_2_O, 10 mM EDTA, and 1 μl of 10 μM unlabelled competitor strand DNA (which has the same sequence as the fluorescent oligodeoxynucleotide) to prevent re-hybridization of the fluorescent products (44). The reaction products were heated to 90°C prior to resolution on a 17% denaturing agarose gel containing 8 M urea; the gel was electrophoresed for 2.5 h at 4 W. Reaction products were visualized using a Typhoon FLA9500 scanner using excitation and emission wavelengths of 542 and 568 nm (TAMRA) and 494 and 520 nm (FITC), respectively.

## RESULTS

### Nuclease properties of *Bl*NucB

**Figure 1.**
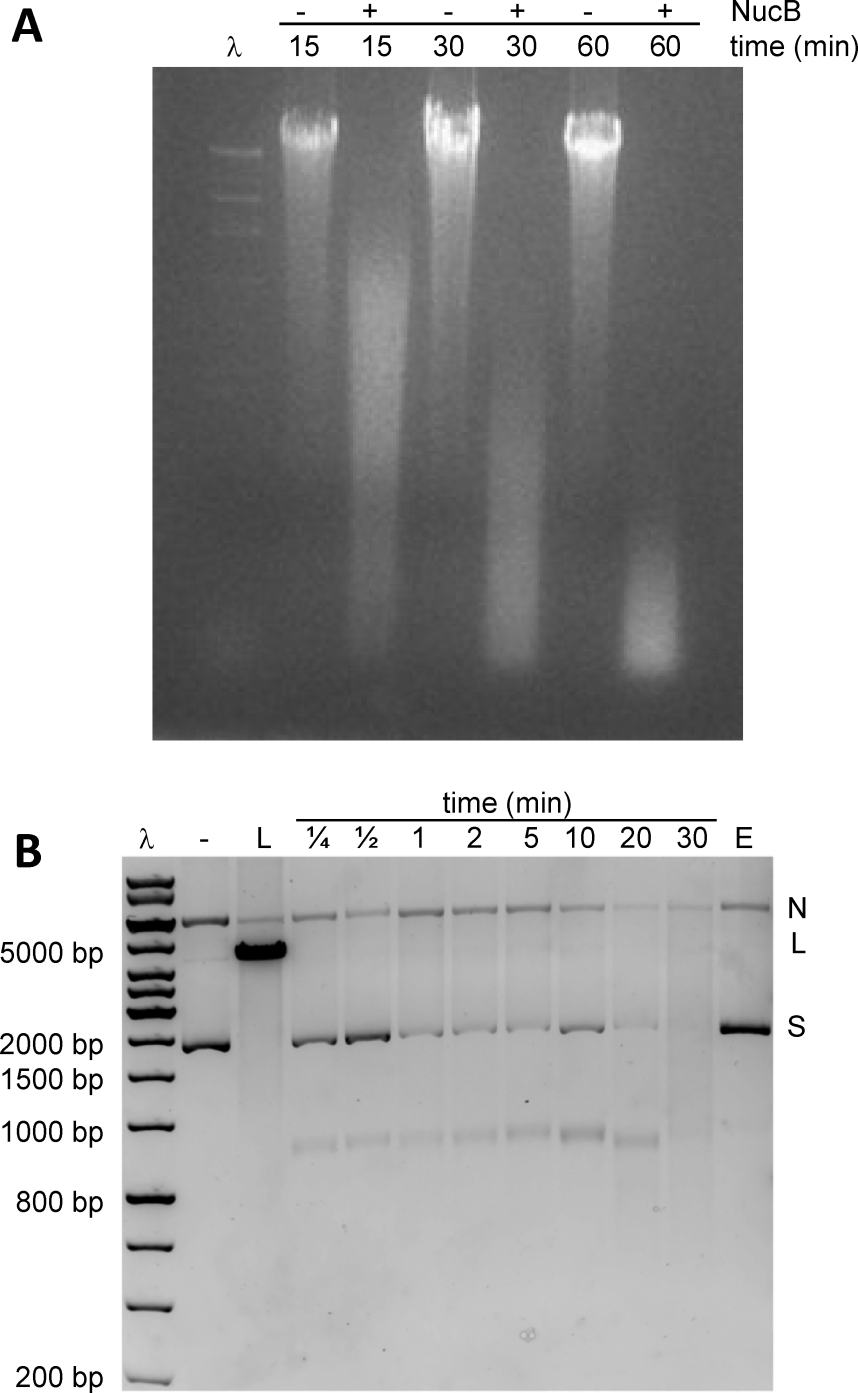
The nuclease activity of *Bl*NucB against high molecular weight DNA. (**A**) *Bl*NucB was incubated with (+) and without (–) calf thymus DNA for 15, 30 and 60 min at 37°C for 1 h. Samples of the digestion products were separated by agarose (0.8% w/v) gel electrophoresis and the DNA made visible by staining with ethidium bromide. HindIII-digested λ DNA (λ) is included as a marker. (**B**) *Bl*NucB was incubated with a mixture of nicked (N) and supercoiled (S) pBR322 DNA at room temperature for 15 s, 30 s, 1 min, 2 min, 5 min, 10 min, 20 min and 30 min (labeled ¼ to 30 min). 10 mM EDTA (E) was added to the reaction mix prior to the addition of *Bl*NucB for 30 min. Undigested (–) and *Bam*HI-linearized (L) pBR322, along with HindIII-digested λ DNA (λ) are included as markers. The digestion products were separated by agarose (0.8% w/v) gel electrophoresis and the DNA made visible by staining with GelRed.

To investigate the activity of *Bl*NucB, its gene was cloned and the recombinant protein was expressed and purified using *B. subtilis* as host. In nuclease assays, 5 ng of *Bl*NucB was capable of digesting 125 μg of calf thymus DNA almost completely in 60 min at 37°C (Figure 1A). Over a 15–60 min time frame, the length of the reaction products was reduced to a minimum, shorter than the 500 bp marker in the *Hin*dIII-digested' phage λ DNA ladder. A semi-quantitative analysis of the degradation of calf thymus DNA by three independent preparations of *Bl*NucB revealed that 0.25 (±0.02; *n* = 9) OD units of acid soluble product was obtained *per* ng of *Bl*NucB *per* hour in buffer containing 5 mM Mn^2+^ ions. Given that double stranded DNA at a concentration of 50 μg/ml has an *A*_260_ of 1 OD units, 1 ng of *Bl*NucB can therefore produce 12.5 μg of low molecular weight DNA per hour.

However, the calf thymus DNA used as the substrate in this experiment is a heterogeneous mix of highly polymerized single and double stranded DNA, and it is thus not possible to determine whether *Bl*NucB acts as an endo- or an exonuclease. To address this question, we determined the effect of *Bl*NucB on supercoiled pBR322 DNA in the presence of Mg^2+^ ions to potentially observe transient intermediates in the reaction trajectory. Since supercoiled DNA does not have free 5′ or 3′ termini, if *Bl*NucB degrades this substrate it must have endonuclease activity. In the first few time points, the bands corresponding to the nicked and supercoiled topoisomers of pBR322 began to disappear such that by 1 min almost all of the supercoiled DNA had either been converted to the nicked form, or had been digested completely (Figure 1B). The substrates were degraded readily in the presence of magnesium, and degradation was inhibited completely in the presence of 10 mM EDTA (Figure 1B). By 30 min, almost all the DNA had been completely degraded. Furthermore, the ∼4.4 kb linear form of the plasmid was not observed in any of the time points, indicating that the DNA was cut one strand at a time. A relatively nuclease-resistant, ∼900 bp product that persisted beyond 20 min, but which was also completely degraded by 30 min, was observed. The nature of this product and its relative stability are unknown. Restriction linearized pBR322 DNA was also degraded completely by *Bl*NucB (data not shown). There was no evidence of the formation of a DNA ladder in any experiment, implying that *Bl*NucB does not have exonuclease activity. The reaction products observed are entirely in keeping with those of a monomeric, metal-dependent, non-specific endonuclease.

**Figure 2.**
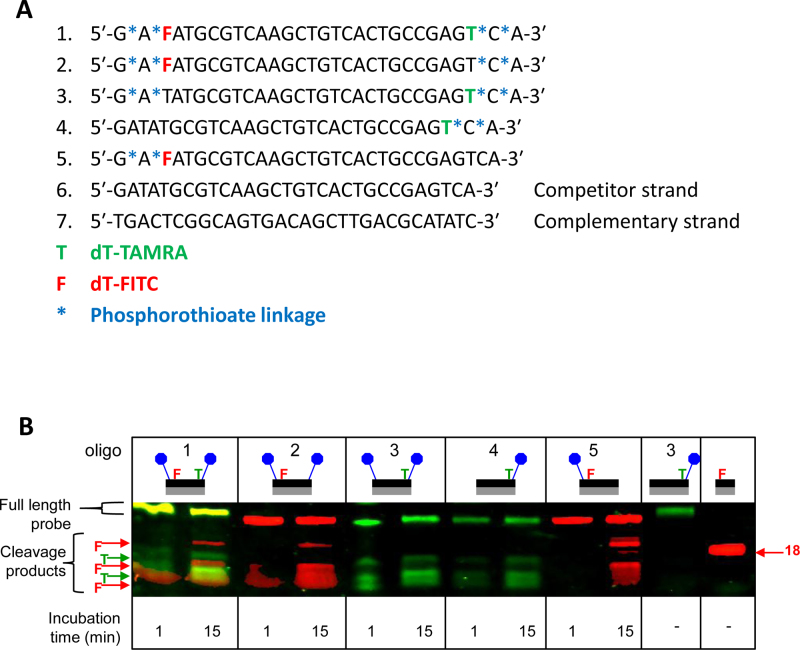
The nuclease activity of *Bl*NucB against oligodeoxynucleotide substrates. (**A**) Sequence of the oligodeoxynucleotides used, with the position of the phosphorothioate linkages indicated with the blue asterisks. (**B**) *Bl*NucB was incubated with fluorescent double stranded oligodeoxynucleotide DNA at room temperature for 1 and 15 min, before separation by 17% (polyacrylamide) denaturing gel electrophoresis and visualization on a Typhoon scanner (GE Healthcare). The non-fluorescent complementary oligodeoxynucleotide used in all reactions is sequence 7. Undigested TAMRA-labelled 30mer and FITC-labelled 18mer oligodeoxynucleotides are used as size controls, and the schematic above the gel indicates which oligodeoxynucleotide was used; phosphorothioates are indicated by the blue circles.

To confirm that *Bl*NucB has solely an endonuclease function, a series of fluorescent oligodeoxynucleotides were synthesized in which either the 5′ or 3′ terminus, or both, was blocked from hydrolysis by the incorporation of an adjacent pair of non-hydrolysable phosphorothioates. If *Bl*NucB had 5′ exonuclease activity, it would not degrade oligodeoxynucleotides substrates blocked at the 5′ end by phosphorothioates, whereas it would remove one base at a time and produce a ladder in the absence of phosphorothioates at the 5′ end. By similar logic, oligodeoxynucleotide substrates blocked at the 3′ end by phosphorothioates would not be a substrate for a 3′ exonuclease. A substrate blocked at both ends could not be hydrolysed by any exonuclease, but it would be hydrolysed as efficiently as a non-blocked substrate by an endonuclease. The sequences of the 30mer oligodeoxynucleotides used in these experiments can be found in Figure 2A.

Representative scans of the in-gel fluorescence from the 3′ TAMRA and/or 5′ FITC fluorescent labels are shown in Figure 2B. There were two timepoints *per* reaction (1 and 15 min) and a FITC-labelled 18mer was included as a marker. Irrespective of the presence or absence of phosphorothioate linkages at the 5′ terminus, there was little to distinguish between the TAMRA-labelled band patterns in any experiment. Similarly, the fluorescence banding patterns from the FITC label was unaffected by the presence of phosphorothioates at the 3′ terminus. No matter how the experiment was conducted, very few products were observed that were larger than 18 bp, and there were no ladders consistent with the removal of a single base at a time from substrates with free termini. Therefore, the DNA was cleaved endonucleolytically, in approximately the centre of the double-stranded 30mer substrate, to produce products in the ∼10mer to ∼20mer range. Close inspection of the resolved fragments from the dual-labelled probe indicates that the oligodeoxynucleotide substrate was cleaved asymmetrically to produce distinct fragments containing either TAMRA- or FITC-labelled (green and red) products. Control experiments with well-characterized exonucleases confirmed that the phosphorothioates protected the termini from degradation (data not shown), and Micrococcal endonuclease also cleaved the dual-labelled probe asymmetrically (Supplementary Figure S1A). In keeping with plasmid DNA as the substrate, the presence of EDTA abrogated endonucleolysis completely (data not shown). When these experiments were repeated with single-stranded DNA substrates, *Bl*NucB cleaved these oligodeoxynucleotides poorly; about half the substrate was cleaved in the first minute and much less cleavage was seen between 1 and 25 min, even when >7-fold more enzyme was included in this reaction (Supplementary Figure S1B). *Bl*NucB cleaved single stranded 30 mer deoxyoligonuclotides at approximately position 20. When the secondary structure of the single stranded substrates were calculated, position 20 tended to occur at the boundary between predicted single and double stranded regions of the DNA. It has already been shown that *Bl*NucB had no detectable activity against total RNA purified from the periodontitis-associated oral bacterium *Veillonella parvula* in comparison to the positive control, RNase A (4). Therefore, *Bl*NucB is a Mn^2+^/Mg^2+^-dependent, non-specific endonuclease that can cleave both single- and double-stranded substrates, but with a preference for double stranded DNA.

### Crystal structure of *Bl*NucB

**Figure 3.**
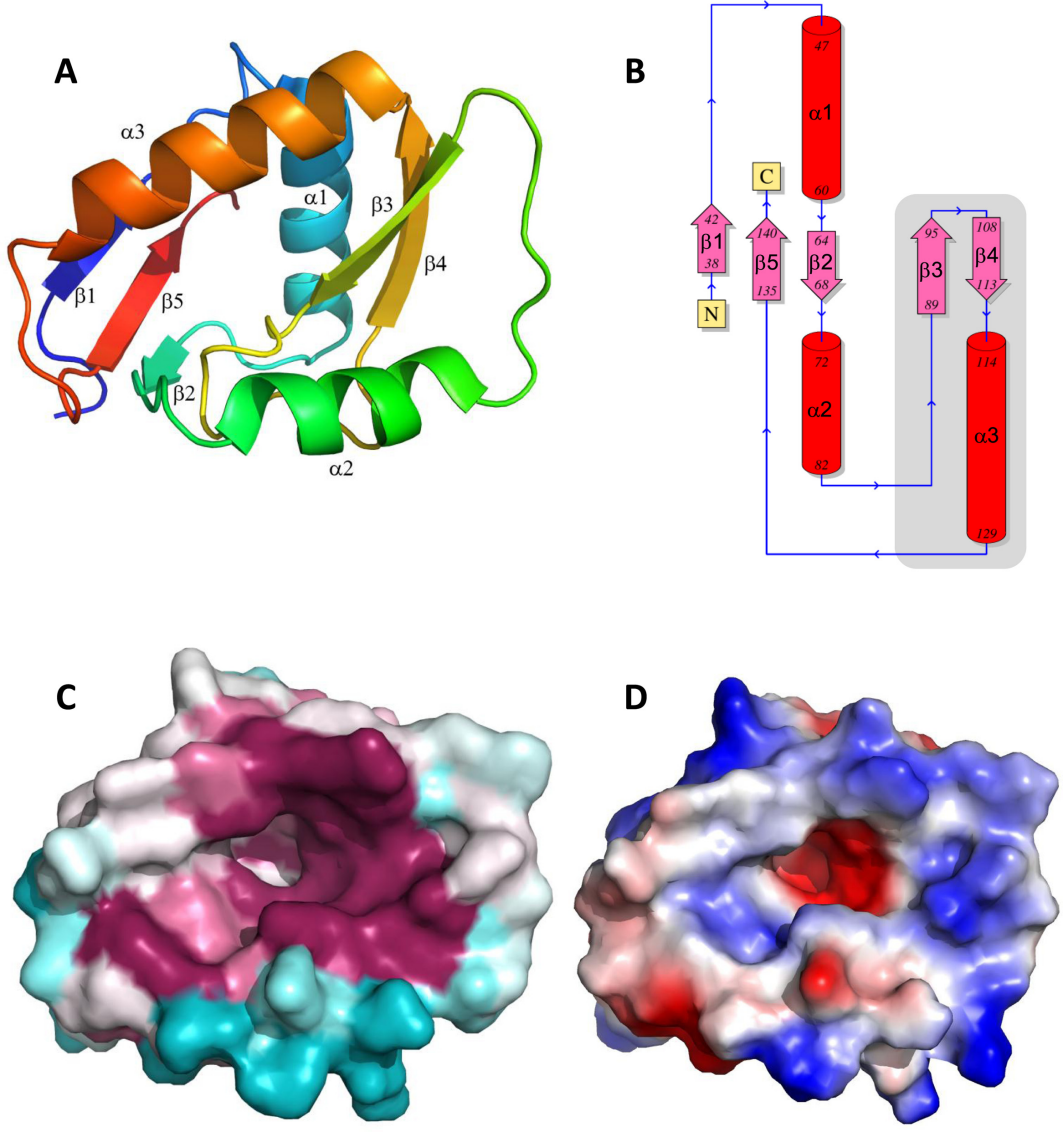
The crystal structure of *Bl*NucB. (**A**) Overview of the structure of *Bl*NucB as a cartoon, colour-ramped from blue to red, from N- to C-terminus. The secondary structure elements are labelled. (**B**) Topology diagram of *Bl*NucB; α-helices are shown as red cylinders and β-strands as pink arrows. The ββα motif that defines the nuclease superfamily to which *Bl*NucB belongs is highlighted by the grey box. (**C**) The surface of *Bl*NucB coloured by sequence conservation (68), drawn in the same view as panel A. Note the concave depression in approximately the middle of the structure the mouth of which is mostly coloured deep purple, which indicates a high degree of sequence conservation. Non-conserved regions are coloured blue, with a purple-blue gradient between the two extremes. (**D**) Electrostatic potential mapped to the surface of *Bl*NucB shown in the same view as above; positively-charged regions are coloured blue, negatively-charged red and uncharged regions are white. Note how the mouth of the depression is positively-charged whereas the bottom of the cavity is negatively-charged.

To understand the molecular basis of its nuclease activity, the structure of *Bl*NucB was solved by single wavelength anomalous dispersion (SAD) from the intrinsic sulphur atoms in the protein and refined to a resolution of 1.35 Å (Supplementary Figure S2). Of the 8594 PDB entries to date that are described under ‘diffraction protocol’ as single wavelength experiments, fewer than 1% have been solved by S-SAD. Careful analysis of highly redundant (58-fold), moderate resolution (2.3 Å), low energy (6.3 keV) diffraction data, collected by rotating a single crystal around one axis, resulted in a straightforward structure solution of a protein with one sulphur atom per 27 residues and a Bijvoet diffraction ratio of ∼1%. Overall, *Bl*NucB is a single, compact domain comprising five β-strands and three α-helices (Figure 3A) that forms a rough triangular pyramid with an approximate diameter of 25 Å. The core of the protein is formed by the five β-strands in a bipartite β-sheet, with β-strands with topological order 1, 5 and 2 separated from β-strands 3 and 4 by the β3-β4 loop that breaks the continuity of the β-sheet (Figure 3B). The β3-β4 loop is stabilized in part by the formation of a disulphide bond between β2 residue Cys66 and the β3-β4 loop residue Cys100 (Supplementary Figure S3). Whilst this disulphide would appear to stabilize the local structure, its reduction with 10 mM DTT had no significant effect on the degradation of calf thymus DNA by *Bl*NucB (data not shown). The base of the pyramid is formed by α-helices 2 and 3 as well as the loop connecting α-helix 2 to β-strand 3, while the peak of the pyramid is formed by the C-terminus of α-helix 1. In keeping with the observed endonuclease activity of *Bl*NucB, the solvent-accessible surface of *Bl*NucB does not reveal a molecular ‘wall’ or ‘tape-measure’ (45) to confer exonuclease activity. Instead, the flat base of the pyramid contains a 15 Å deep, 10 Å wide, 20 Å long concave depression that is formed mostly by conserved amino acids (Figure 3C). The base of the depression is predominantly negatively-charged, whereas the lips of the cavity are mostly positively-charged (Figure 3D). The surface depression is of sufficient size to accommodate a single strand of DNA, presenting the scissile phosphodiester bond to the catalytic apparatus, whilst the lips of the cavity can interact with the DNA phosphate backbone.

**Figure 4.**
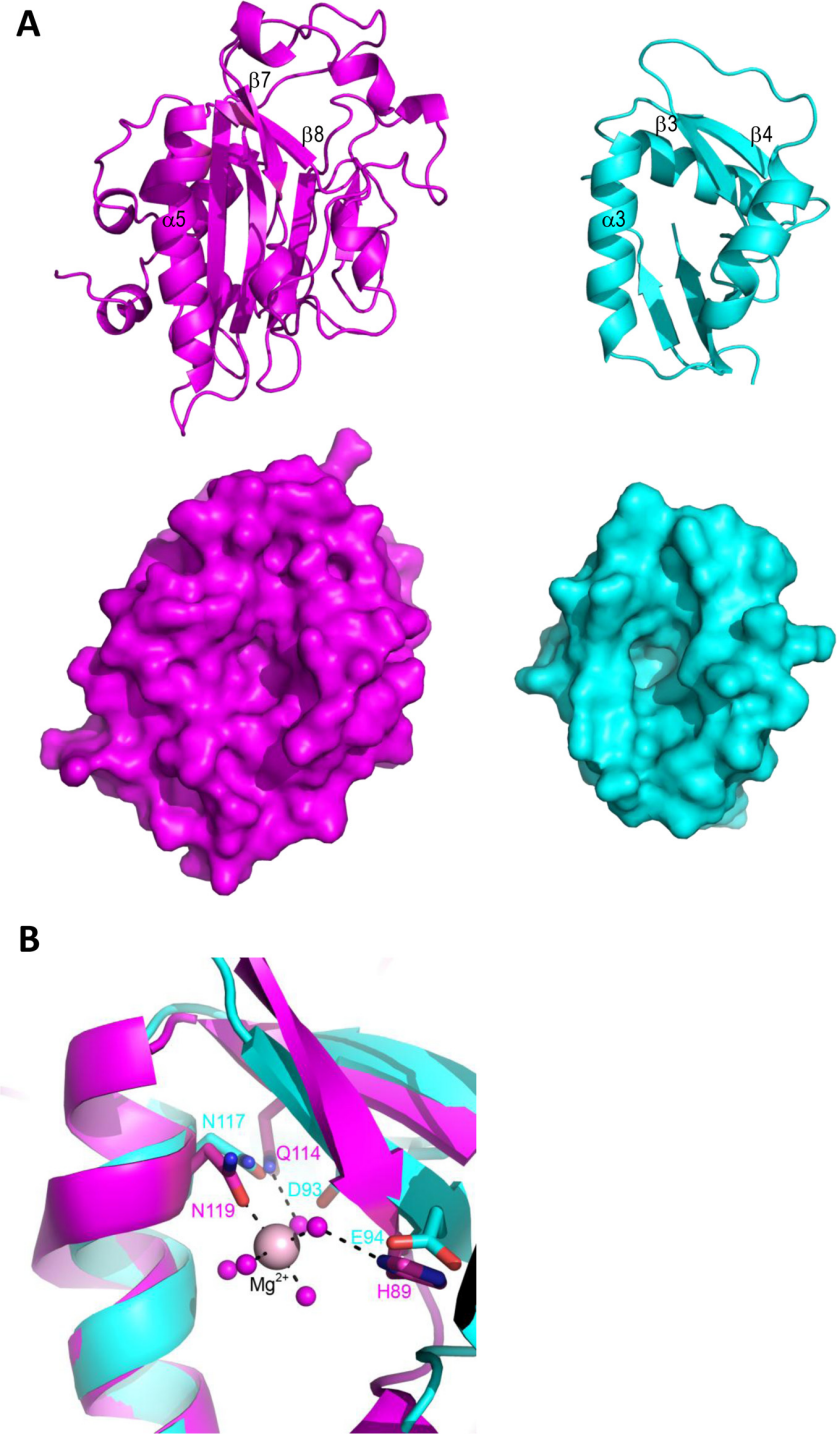
*Bl*NucB belongs to the ββα family of non-specific endonucleases. (**A**) The structure of *Bl*NucB (cyan) indicates that it belongs to a novel subgroup of the ββα (or His-Me finger) family of non-specific endonucleases, as exemplified by *Sm*endo (magenta; PDBid: 1SMN; 45). The ββα motif that defines the active site is labelled in each structure by the corresponding secondary structure element (top row). Other than this motif, the structural homology between *Bl*NucB and *Sm*endo is not extensive, but the surface representation in the same view (bottom row) indicates that they share the same surface depression that houses the active site. (**B**) A superposition of the active sites of *Sm*endo (magenta) vs *Bl*NucB (cyan), showing the conservation of the metal binding site in the latter. Asn119 in *Sm*endo forms the only direct proteinaceous contact to the bound Mg^2+^ ion (pale pink sphere), and the water shell around the metal ion is stabilized by interactions to Gln114 and the general base, His89. The structural equivalents in *Bl*NucB are Asn117, Asp93 and Glu94, respectively. Residues are coloured by structure, cyan for *Bl*NucB and magenta for *Sm*endo.

Though NucB orthologues appear to form a unique protein family by sequence, searches of the PDB with the atomic co-ordinates of *Bl*NucB revealed that it does have some structural homology to the *Serratia marcescens* endonuclease (*Sm*endo; PDBid: 1SMN; 46), with an RMSD of 3.6 Å on 77 superimposed Cαs (Figure 4A). At first glance, the homology between *Sm*endo and *Bl*NucB is not readily apparent: *Sm*endo is approximately twice the size of *Bl*NucB (245 residues *vs* 109), but the protein core and functionally essential structural elements are maintained (see below; Figure 4A). *Sm*endo is a member of the His-Me finger family of endonucleases, where Me stands for a divalent cation and where His refers to the general base in the reaction (47). The magnesium ion in *Sm*endo is co-ordinated directly by only one protein atom, the sidechain amide oxygen of Asn119, and the rest of the magnesium's hydration shell is filled by water molecules, one of which is co-ordinated by the mainchain amide and sidechain Nδ1 nitrogen from the His89 general base (Figure 4B). This arrangement is mostly, but not completely, maintained in other His-Me finger endonucleases; in some instances, including the *Vibrio vulnificus* endonuclease (*Vvn*endo), there is a direct contact to the cation from an acidic residue, Glu79, located immediately prior to the general base, His80 (47,48). *Sm*endo has glycine (Gly88) adjacent to the His89 general base, explaining why the sole protein contact to the cation in this protein comes from Asn119 (46). There is no metal ion bound in the apo structure of *Bl*NucB reported here and, despite repeated attempts, we were unable to obtain diffracting crystals containing either bound divalent cations or oligodeoxynucleotides. As with other members of the His-Me finger family, *Bl*NucB retains the metal-binding pocket (Figure 4B), including the metal-chelator Asn117 and, as in *Vvn*endo (48), *Bl*NucB encodes an acid (Asp93) immediately before Glu94 and located so that with minimal changes to the sidechain chi angles it can be positioned to co-ordinate directly to a bound divalent cation. The His-Me finger family is also known as the ββα family, which relates to the maintenance of centrally-positioned secondary structural elements that harbour the essential catalytic residues (47). The ββα nomenclature is adopted here in relation to *Bl*NucB because it lacks the conserved histidine general base of the His-Me finger family, which has been replaced in *Bl*NucB by Glu94, and thus NucB is the founding member of a novel subgroup of the His-Me finger family of non-specific endonucleases.

### The NucB active site

**Figure 5.**
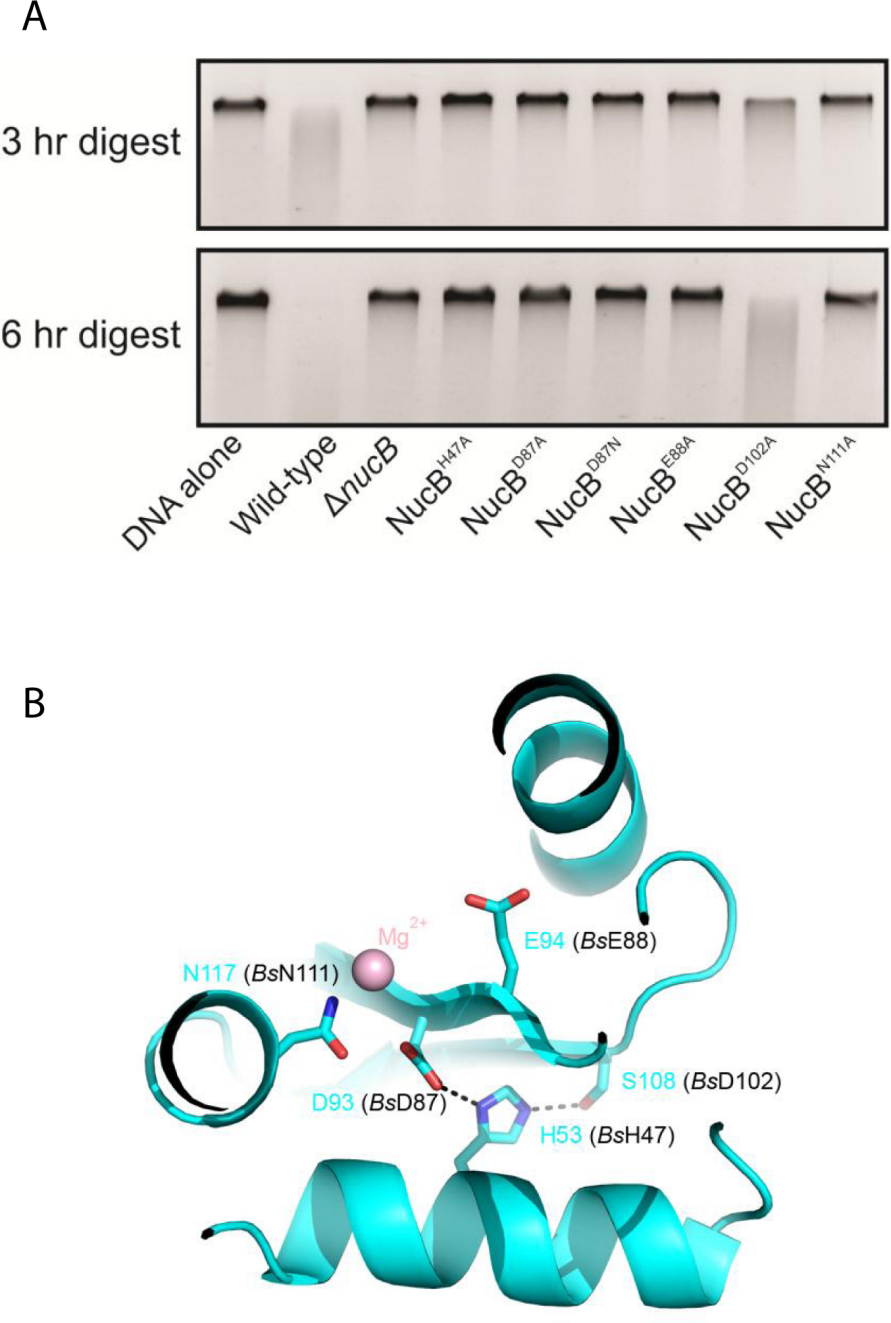
NucB nuclease activity *ex vivo*. (**A**) *Bs*NucB mutants are defective in nuclease activity. Cells were grown in Schaeffers media to induce sporulation at 37°C. Supernatant were then mixed with chromosomal DNA in the ratio 1:3 for 3 and 6 h at 37°C before DNA was visualized using 1% agarose gel stained with SYBR Gold. Wild-type (HM715), Δ*nucB* (AK361), *nucB*^H47A^ (AK453), *nucB*^D87A^ (AK447), *nucB*^D87N^ (AK449), *nucB*^D88A^ (HM1766) *nucB*^D102A^ (AK451), *nucB*^N111A^ (HM1767). (**B**) The active site of *Bl*NucB is shown after rotation of the view in Figure 4A around a horizontal axis of ∼90°. The equivalent residues in *Bl*NucB that were mutated in *Bs*NucB (Figure 5A) are labelled in cyan; their counterparts in *Bs*NucB are additionally labelled in black. Though *Bs*NucB His47 is removed from the active site, mutation of it and immediate neighbours presumably affects nuclease activity because of the loss of a stabilizing network of hydrogen bonds involving Asp87 and Asp102; the equivalents in *Bl*NucB are His53, Asp93 and Ser108.

In order to confirm the importance of likely active centre residues of *Bl*NucB, the nuclease activity of several variants (Figure 5A, B) was analyzed *ex vivo* using *B. subtilis*, in which we have an established genetic system. The amino acid sequence homology between mature NucB orthologues from *B. subtilis* and *B. licheniformis* is 77% (Supplementary Figure S3), and all the metal-binding and proposed catalytic residues are conserved, indicating that functional data from one system will be directly relevant to structural and biochemical data in the other, and *vice versa*. Wild-type *BsnucB* was replaced by mutant alleles whilst retaining the native expression system and secretion signal. Cultures were grown into stationary phase to induce *BsnucB* expression and supernatants containing the secreted *Bs*NucB proteins were collected. To assess overall nuclease activity the supernatants were incubated with purified chromosomal DNA. Subsequently the genomic DNA fragments were stained with a fluorescent dye, resolved using agarose gel electrophoresis, and visualized by UV transillumination (Figure 5A). Whilst the supernatant from the wild-type strain led to the digestion of the DNA substrate, the supernatants from strains expressing *Bs*NucB^H47A^, (equivalent to *Bl*NucB^H53^), *Bs*NucB^D87A^ and *Bs*NucB^D87N^ (*Bl*NucB^D93^), *Bs*NucB^E88A^ (*Bl*NucB^E94^), and *Bs*NucB^N111A^ (*Bl*NucB^N117^), in addition to the Δ*BsnucB* mutant, showed no significant nuclease activity. In *Bl*NucB, Asp93 is likely to stabilize the bound magnesium ion that is essential for nuclease activity most likely by co-ordinating directly to it. Alternatively, Asp93 might bind indirectly to the magnesium via a bridging water molecule, but this is less likely because of steric clashes that would result between Asp93 and His53 by introducing such a water molecule. Replacement of this aspartate with alanine will clearly disrupt the magnesium co-ordination potential of NucB, most likely a direct contact given the complete loss of nuclease activity associated with mutations at this allele and the steric clashes that would likely occur by the introduction of a bridging water molecule. The importance of the correct positioning of the ion for catalysis is underlined by the loss of activity associated with the *Bs*NucB^H47A^ allele, since the *Bl*NucB^H53^ equivalent maintains the position of Asp93 by a hydrogen bond network in a manner that is reminiscent of a catalytic triad (Figure 5B). The third member of this triad, *Bl*NucB^S108^, is positioned on the other side of His53 and mutation of the non-conserved equivalent, *Bs*NucB^D102^ also has a negative impact on *Bs*NucB activity. To confirm the importance of Asp93 for activity, the asparagine variant at this position was introduced to *Bl*NucB by site directed mutagenesis and the protein purified by the same protocol as for the wildtype protein. In keeping with these data and the importance of Asp93 for co-ordinating the catalytically-essential metal ion, the *Bl*NucB^D93N^ variant had less than 1% of the wild type activity in semi-quantitative assays based on the degradation of calf thymus DNA (Supplementary Figure S4A). Purified *Bl*NucB preparations showed a single protein band when increasing loadings were analysed by SDS PAGE and spectroscopic analysis confirmed the absence of nucleic acid. (4). Peptide mass fingerprinting of a trypsin digest of the single protein band excised from the SDS PAGE gel showed that the only protein present was *Bl*NucB (4). Furthermore, purified *Bl*NucB preparations had no detectable RNase activity against total bacterial RNA in comparison to RNase A and no detectable protease activity against BSA in comparison to proteinase K (4). These observations, combined with the quantitative loss of over 99% of the nuclease activity of the *Bl*NucB^D93N^ variant compared to the wildtype *Bl*NucB when purified under the same conditions (Supplementary Figure S4A), argues strongly that the nuclease activity seen in Figures 1 and 2 is due to the wildtype *Bl*NucB present and not a co-purifying contaminant.

### Thermal stability of *Bl*NucB

The NucB family is exemplified by its small size; the mature enzyme is only 110 amino acids in length, approximately half the size of the crystallized secreted endonucleases from *Anabaena* (e.g. PDBid: 1ZM8), *Streptococcus agalactiae* (e.g. PDBid: 4QGO) and *Serratia marcescens* (e.g. PDBid: 1SMN), and half the size of the recombinant human DNase I used to treat cystic fibrosis. NucB is slightly bigger than the catalytic domain (∼95 residues) of the GIY-YIG endonuclease family, but unlike NucB, GIY-YIG family members are modular, with separate DNA-binding and nuclease domains though the isolated nuclease domain retains the ability to bind to and degrade DNA non-specifically (47). In the context of the potential biotechnological exploitation of NucB, the thermal stability of *Bl*NucB was investigated by DSC. In the presence of 1 mM DTT, *Bl*NucB had a single unfolding event with a *T*_m_ of 57.4 (±0.1)°C (Supplementary Figure S4B). When fully unfolded protein was allowed to cool passively to 25°C, and then subjected to a second round of thermal unfolding, a *T*_m_ of 58.7 (±0.1)°C was obtained (Supplementary Figure S4B). The calculated enthalpy of the first unfolding event was 71 (±10.4) kcal mol^−1^, whereas for the second unfolding event the enthalpy was 37 (±5.2) kcal mol^−1^, indicating that 52% of the *Bl*NucB sample refolded spontaneously when cooled passively to 25°C. Semi-quantitative nuclease assays using *Bl*NucB that had been subjected to thermal unfolding and then refolded passively showed that the refolded protein had regained approximately 36% of its activity in comparison to unheated samples (Supplementary Figure S4C). Hence, native *BlN*ucB can be thermally unfolded in the presence of reducing agents, refold spontaneously and regain significant nuclease activity, suggesting that forced evolution of *Bl*NucB could improve these biotechnologically desirable characteristics even further.

## DISCUSSION

The *Bl*NucB protein sequence does not identify other nuclease families when used to query the BLAST non-redundant sequence database. Although some *de novo* structure predictions could recapitulate the overall fold of *Bl*NucB, the models were insufficiently accurate to identify the active site or the nuclease family to which *Bl*NucB belongs (49), and molecular replacement using these models was unsuccessful. The structure of *Bl*NucB was instead solved by S-SAD without recourse to sophisticated data collection strategies. Perhaps, as advocated elsewhere (50), S-SAD has greater latent potential for solving the crystallographic phase problem than appears to be generally appreciated.

Consequent structure-based searches of the PDB revealed that *Bl*NucB belongs to the divergent His-Me finger family of endonucleases (47), despite sharing less than 12% sequence identity with its closest structural neighbour, the endonuclease from *Serratia marcescens, Sm*endo. Structures of several representatives of the His-Me finger nucleases have been solved (48,51–54), which are involved in diverse cellular functions (47) including, for example, secreted toxins (51,52), protection from transformation by extracellular DNA (48), Holliday junction resolution (53), and prophage induction (54). NucB, on the other hand, is necessary for the degradation of eDNA for the dispersal of bacterial biofilms (27). Most His-Me finger nucleases cleave DNA non-specifically (47), and some have been shown to also work on single-stranded DNA and RNA (47). *Bl*NucB is a non-specific endonuclease, which can hydrolyse both single- and double-stranded DNA substrates, as determined by its ability to completely hydrolyse supercoiled plasmid DNA substrates that have no free 3′ or 5′ termini (Figure 1B), and to degrade short oligonucleotides whose 5′ and 3′ termini are blocked because they were synthesized with non-hydrolysable phosphorothioates (Figure 2A, B). Since both single- and double-stranded DNA play roles in biofilm formation (55) it is entirely appropriate that NucB has evolved to disperse biofilms with precisely these properties.

His-Me finger nucleases generally utilize a single metal ion as a Lewis acid and a histidine general base for hydrolysis of the phosphodiester DNA backbone, and are characterized by a common ββα motif (47). *Bl*NucB diverges from this general description because glutamate replaces the histidine, and this glutamate is essential for biological activity (Figure 5A); therefore *Bl*NucB is the founding member of a novel subfamily of the His-Me finger nuclease superfamily. The histidine general base in colicin E7 (His545) and I-PpoI (His98) has each been mutated to glutamate, but the resultant enzymes lose biological activity almost entirely (56,57), indicating that other facets of the NucB active site must compensate for the absence of the histidine, but it is not immediately apparent from the *Bl*NucB structure what this facet could be. The activity of *Bs*NucB has already been measured as a function of pH and appears to show a bell-shaped pH dependency with a pH optimum of 7.5 (24). The apparent bell-shaped curve could suggest the involvement of two amino acids in catalysis with p*K*_a_s above and below neutral. Presumably one of these amino acids is Glu94, but it is not readily apparent from the structure of *Bl*NucB what the other ionizable group could be. Alternatively, the rapid drop-off in activity seen at pH 8 and above might reflect the precipitation of Mn(OH)_2_ in mildly alkaline conditions with a concomitant reduction in enzyme activity—as observed previously for *Anabaena* NucA (58). The drop-off in enzyme activity with increase in pH may also reflect the partial destruction of the disulphide by base-catalysed β-elimination, though this seems unlikely given the retention of enzyme activity in 10 mM DTT, sufficient to reduce the disulphide. The ββα motif contains a central pair of anti-parallel β-strands flanked by a conserved distal α-helix that sits in the major groove of DNA; the loop connecting the two β-strands is variable in length and conformation. The histidine general base (46,47,59) is located in the middle of the first of the two β-strands of the ββα motif, and does not interact directly with the bound nearby catalytic cation. Instead, the cation is co-ordinated directly usually by an asparagine found towards the N-terminus of the conserved distal α-helix, and this is the only direct contact between protein and cation in *Sm*endo (46) because a glycine is found at the structurally equivalent position to the glutamate and aspartate found in *Vvn*endo (48) and NucB, respectively. Histidine is required to co-ordinate the cation in zinc-dependent His-Me finger nucleases like ColE7 (51) and ColE9 (52), whereas the magnesium- and manganese-dependent enzymes require asparagine or aspartate (e.g. 43,48,53,60). In the absence of a structure of *Bl*NucB with a bound cation, and based on the structural similarity of *Bl*NucB to magnesium-dependent His-Me finger nucleases, the co-ordination sphere of the bound metal ion is likely to be fulfilled by the sidechain amide oxygen of Asn117, the sidechain carboxylate of Asp93 and four water molecules, some which could be stabilized by their contacts to the sidechains oxygens of Asp93 and Glu94, and mainchain atoms from Asp93, Glu94 and Asn117 (Figure 4B).

The similarity of the catalytic apparatus of *Bl*NucB to His-Me finger-type endonucleases is underlined by the structural superposition of the core ββα motif of *Bl*NucB to that found in the structure of, for example, the *Vvn* endonuclease (48). The rmsd of the matched motifs is 0.7 Å, and this superposition places all the catalytic machinery of *Bl*NucB in equivalent positions to those found in *Vvn*, and the metal ion and its co-ordinating waters that is present in the structure of *Vvn*, but which are absent from the structure of *Bl*NucB, into positions that are consistent with roles in catalysis in *Bl*NucB. Furthermore, the key amino acids (underlined) for direct magnesium co-ordination are highly conserved in NucB sequences in DRDE and DNRG motifs (Supplementary Figure S5) on the second β-strand and the α-helix, respectively, of the ββα sub-structure. The importance of these sequence motifs to metal-binding and catalysis is emphasized by the observation that mutation of individual amino acids in these motifs in *Bs*NucB (Asp93 and Glu94 from motif 1, and Asn117 from motif 2) abrogates enzyme activity completely (Figure 5A).

**Figure 6.**
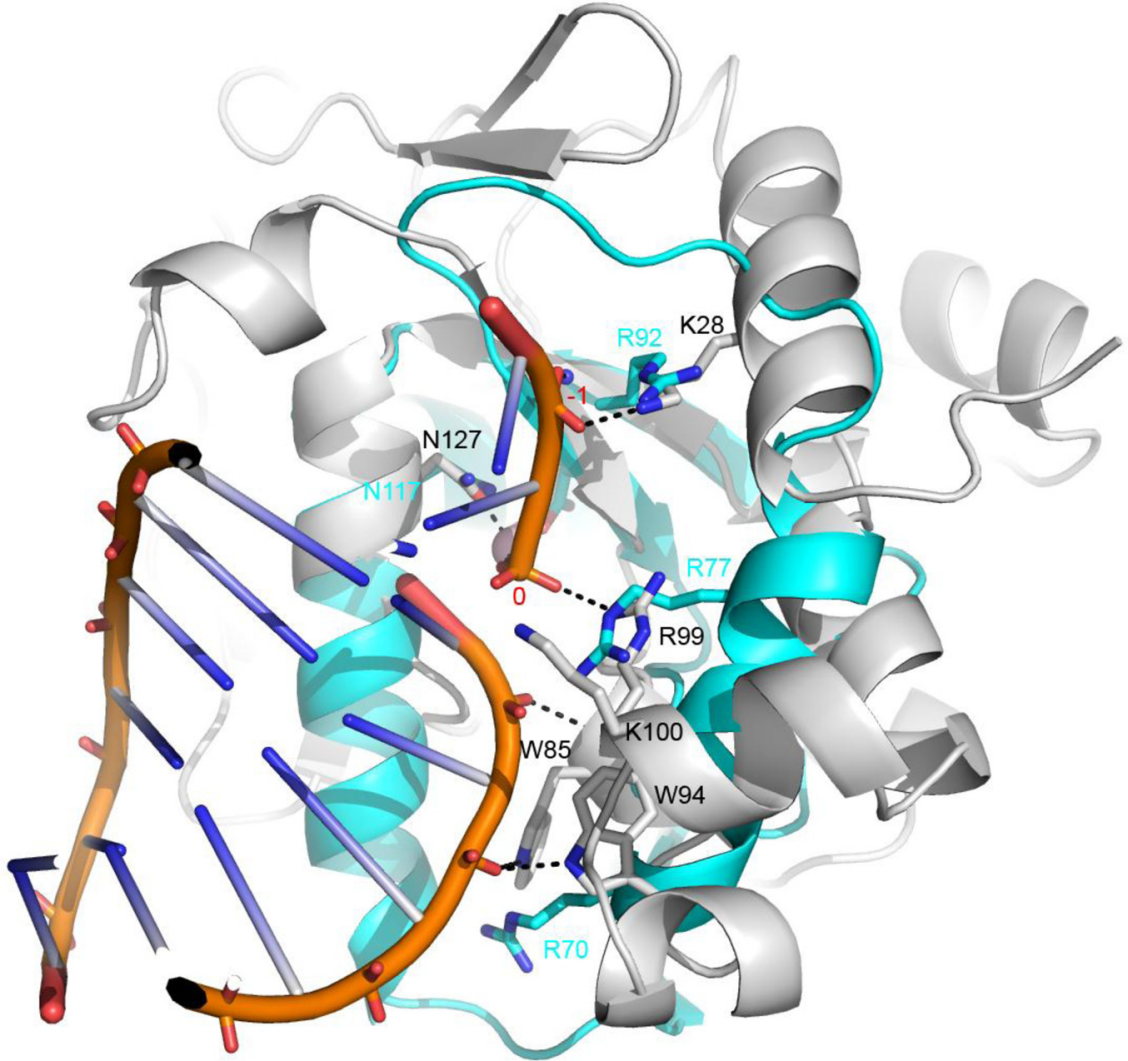
A model of the interaction of *Bl*NucB with DNA. A model for the interaction of *Bl*NucB (cyan) with post-cleavage DNA (orange worm) based on the shared structural feature of the ββα motif in *Vvn* endonuclease (silver; PDBid: 1OUP; 52). Key amino acids for *Vvn* DNA recognition are shown as sticks and are labelled in silver K28, E79, R99 and N127, with cyan labelled *Bl*NucB structural equivalents R92, D93, R77 and N117. *Vvn* K100 does not seem to have a structural equivalent in *Bl*NucB and the interaction of *Vvn* residues W85 and W94 with the DNA backbone remote from the active site is likely performed by *Bl*NucB R70. The scissile phosphate is labelled with a red ‘0’ and the –1 phosphate is also indicated.

In common with most other non-specific nucleases, including human DNase I (61), it is likely that NucB interacts with its double stranded substrates in the DNA minor groove (Figure 6). Base-specific interactions tend to require access to the major groove, which is not necessary for non-specific DNA interactions (62). For instance, there are only two base specific contacts in the structure of the non-specific *Vvn* endonuclease in the presence of DNA (48). Many of the amino acids used by *Vvn* to interact with DNA are conserved spatially in *Bl*NucB and superimpose closely (Figure 6). For instance, the phosphate at –1 in the *Vvn*:DNA complex (48) is contacted by the Nζ atom of Lys28, the equivalent residue in *Bl*NucB is Arg92. The sidechain of Asn127 of *Vvn* interacts with the scissile phosphate and its O5′ oxygen; Asn117 is the equivalent residue in *Bl*NucB. The scissile phosphate in *Vvn* is also contacted by the guanidinium group of Arg99, which overlaps with that of *Bl*NucB Arg77 despite the 8 Å separation of their respective Cα atoms. These arginine residues probably play critical roles in their respective proteins by stabilizing the developing negative charge on the phosphate in the transition state.

*B. subtilis* encodes another glutamate-dependent variant of the ββα endonuclease family with 78% sequence identity to NucB; the gene for this nuclease, NucA, is found adjacent on the chromosome to that of the presumed nuclease inhibitor, Nin. When lysates were mixed from *B. subtilis* strains that lacked either *nin, nucA* or *nucB*, Nin was capable of inhibiting both NucA and NucB in nuclease assays (63). Does the structure of *Bl*NucB provide insight into nuclease inhibition by Nin? A crystal structure of NucA in complex with its cognate inhibitor, NuiA (Supplementary Figure S6A), has been solved from the cyanobacterium, *Anabaena* (61). Though *Anabaena* NucA and *Bl*NucB share no significant sequence homology, *Anabaena* NucA is also a member of the His-Me finger family of nucleases. *Anabaena* NuiA is a competitive inhibitor of *Anabaena* NucA and binds to the nuclease in a site that overlaps with that of the substrate DNA (60). However, Nin and NuiA share no meaningful sequence homology and inspection of their structures (the unpublished structure of Nin has been determined by a structural genomics consortium, PDBid: 4MQD) reveals that they also share little structural homology (Supplementary Figure S6B). It thus remains to be seen how Nin exerts its inhibitory effect on NucA and/or NucB.

Finally, various properties of *Bl*NucB are pertinent to its biotechnological potential in biofilm dispersal (28). *Bl*NucB is a potent, non-specific endonuclease that can degrade double- and single-stranded DNA and topologically complex molecules, such as supercoiled DNA. *Bl*NucB is a thermally robust enzyme that regains nuclease activity after a heat-cool cycle (Supplementary Figure S4B, C). By contrast, the recombinant human DNase I used in treatments for cystic fibrosis requires two N-linked glycosylation sites for full enzyme activity, thermal stability and protease resistance (64); moreover, human DNase I does not refold spontaneously after thermal denaturation (65). Possible methods to further enhance the *in vivo* allergenicity, activity and stability of *Bl*NucB to address the health and societal challenges raised by biofilms include gene site saturation mutagenesis and/or the introduction of novel disulphides; these methods have found utility in improving xylanases for paper pulping (66) and viruses for vaccine production (67). Future work in our laboratories will focus on the precise roles played by the various elements of the catalytic machinery, determining how NucA and NucB utilize glutamate in catalysis, and how Nin exerts its inhibitory effects on NucA and NucB.

## AVAILABILITY

The structure of NucB has been deposited in the Protein Data Bank (http://www.rcsb.org/pdb/home/home.do) under accession number 5OMT.

## Supplementary Material

Supplementary Data
