# Supplementary material for: Crystal structure of NucB, a biofilm-degrading endonuclease
Source: Nucleic Acids Res. 2017 Nov 20;46(1):473–84. doi: 10.1093/nar/gkx1170 (PMC5758888; doi:10.1093/nar/gkx1170)
Supplement: Supplementary Data [file gkx1170_supp.docx]

Supplementary Information for:

**Crystal structure of NucB, a biofilm-degrading endonuclease**

Arnaud Baslé^1#^, Lorraine Hewitt^1#^, Alan Koh^2,5#^, Heather K. Lamb^1#^, Paul Thompson^1^, J. Grant Burgess^3^, Michael J. Hall^4^, Alastair R. Hawkins^1^, Heath Murray^2^, Richard J. Lewis^1^*

^1^Institute for Cell and Molecular Biosciences, Faculty of Medical Sciences, Newcastle University, Newcastle upon Tyne, NE2 4HH, UK

^2^Centre for Bacterial Cell Biology, Faculty of Medical Sciences, Newcastle University, Newcastle upon Tyne, NE2 4AX, UK

^3^Marine Biology, School of Natural and Environmental Sciences, Newcastle University, Newcastle upon Tyne, NE1 7RU, UK

^4^Chemistry, School of Natural and Environmental Sciences, Newcastle University, Newcastle upon Tyne, NE1 7RU, UK

*For correspondence: Richard J. Lewis, Institute for Cell and Molecular Biosciences, Faculty of Medical Sciences, Newcastle University, Newcastle upon Tyne, NE2 4HH, UK, tel: +44 (0)191 208 5482, email: [r.lewis@ncl.ac.uk](mailto:r.lewis@ncl.ac.uk)

^#^Contributed equally

^5^Current address: Alan Koh, Milner Centre for Evolution, Department of Biology and Biochemistry, University of Bath, BA2 7AY, UK

Key words: biofilm / X-ray crystallography / endonuclease

**Supplementary methods**

**Plasmid construction**

pAK91 [*bla cat yqeB^ATG→TAA^ zeo nucB*^ATG^*^→^*^TAA^] was generated by ligation with a *Sac*I-*Sac*II PCR product containing *nucB* with the start codon changed to a stop codon (oAK74 + oAK101 and 168CA genomic DNA as template) and pAK316 cut with *Sac*I-*Sac*II.

pAK108-pAK111 and pHM629-pHM630 were generated by site-directed mutagenesis using primers listed in **Supplementary Table 3** using pAK91 [*bla cat yqeB^ATG→TAA^ zeo nucB*^ATG^*^→^*^TAA^] as template.

pAK184-pAK186 were generated by site-directed mutagenesis using primers listed in **Supplementary Table 3** using pNZ8901, containing *nucB* from *B. licheniformis* as a Eco91I/XbaI fragment [*cat* P*_spaS_*_mut_*-nucB*] as template.

pAK316 [*bla cat yqeB^ATG→TAA^ zeo*] was generated by ligation with a *Apa*I-*Cla*I PCR product containing *yeqB* with the start codon changed to a stop codon (oAK76 + oAK77 and 168CA genomic DNA as template) and pHM457 (1) cut with *Apa*I-*Cla*I.

pEM5 [*bla cat yqeB*^ATG^*^→^*^TAA^ *neo* P*_soj_*-*soj-spo0J*] was generated by ligation with a *Sac*I-*Not*I PCR product containing *yeqB* with the start codon changed to a stop codon (oAK71 + oAK72 and 168CA genomic DNA as template) and pHM16 (2) cut with *Sac*I-*Not*I.

pEM6 [*bla cat yqeB*^ATG^*^→^*^TAA^ *neo nucB’ spoIVCA*] was generated by ligation with a *Xho*I-*Spe*I PCR product containing the *spoIVCA* and 5′ end of *nucB* (oAK69 + oAK70 and 168CA genomic DNA as template) and pEM05 cut with *Xho*I-*Spe*I.

**Supplementary references**

1. Marbouty M, Le Gall A, Cattoni DI, Cournac A, Koh A, Fiche JB, Mozziconacci J, Murray H, Koszul R, Nollmann M. (2015) Condensin- and replication-mediated bacterial chromosome folding and origin condensation revealed by Hi-C and super-resolution imaging. *Mol Cell* **59**, 588-602.

2. Murray H, Ferreira H, Errington J. (2006) The bacterial chromosome segregation protein Spo0J spreads along DNA from *parS* nucleation sites. *Mol Microbiol* **61**, 1352-1361.

3. Kunst F and 149 others (1997) The complete genome sequence of the Gram-positive bacterium *Bacillus subtilis*. *Nature* **390**, 249-256.

4. Bongers RS, Veening JW, Van Wieringen M, Kuipers OP, Kleerebezem M. (2005) Development and characterization of a subtilin-regulated expression system in *Bacillus subtilis*: strict control of gene expression by addition of subtilin. *Appl Environ Microbiol* **71**, 8818-8824.

5. Nijland N, Hall MJ, Burgess JG. (2010) Dispersal of biofilms by secreted, matrix degrading, bacterial DNase. *PLoS One* **5**, e15668.

6. Ghosh M, Meiss G, Pingoud AM, London RE, Pedersen LC. (2007) The nuclease a-inhibitor complex is characterized by a novel metal ion bridge. *J Biol Chem* **282**, 5682–5690.

**Supplementary Table 1: Strain list.**

| **Strain** | **Genotype** | **Parent strain** | **Plasmid used** | **Reference** |
| --- | --- | --- | --- | --- |
| AK361 | *trpC2 ∆nucB*::*neo* | HM715 | pEM6 | this work |
| AK447 | *trpC2 nucB*^D87A^::*zeo* | HM715 | pAK108 | this work |
| AK449 | *trpC2 nucB*^D87N^::*zeo* | HM715 | pAK109 | this work |
| AK451 | *trpC2 nucB*^D102A^::*zeo* | HM715 | pAK110 | this work |
| AK453 | *trpC2 nucB*^H47A^::*zeo* | HM715 | pAK111 | this work |
| AK659 | *trpC2 amyE*::*neo*(*spaRK*) (P*_spaS_*_mut_-*BlnucB*^H53A^)*cat* | NZ8900 | pAK184 | this work |
| AK661 | *trpC2 amyE*::*neo*(*spaRK*) (P*_spaS_*_mut_-*BlnucB*^D93A^)*cat* | NZ8900 | pAK185 | this work |
| AK663 | *trpC2 amyE*::*neo*(*spaRK*) (P*_spaS_*_mut_-*BlnucB*^D93N^)*cat* | NZ8900 | pAK186 | this work |
| HM715 (168CA) | *trpC2* |  |  | 3 |
| HM1766 | *trpC2 nucB*^E88A^::*zeo* | HM715 | pHM629 | this work |
| HM1767 | *trpC2 nucB*^N111A^::*zeo* | HM715 | pHM630 | this work |
| NZ8900 | *trpC2 amyE*::*neo*(*spaRK*) |  |  | 4 |

**Supplementary Table 2: Plasmid list.**

| **Plasmid** | **Genotype** | **Parent plasmid** | **Oligos used for construction** | **Reference** |
| --- | --- | --- | --- | --- |
| pAK91 | *bla cat yqeB*^ATG→TAA^ *zeo nucB*^ATG→TAA^ | pAK316 | oAK74/oAK101 | This work |
| pAK108 | *bla cat yqeB*^ATG→TAA^ *zeo nucB*^ATG→TAA,D87A^ | pAK91 | oAK102/oAK103 | This work |
| pAK109 | *bla cat yqeB*^ATG→TAA^ *zeo nucB*^ATG→TAA,D87N^ | pAK91 | oAK104/oAK105 | This work |
| pAK110 | *bla cat yqeB*^ATG→TAA^ *zeo nucB*^ATG→TAA,D102A^ | pAK91 | oAK106/oAK107 | This work |
| pAK111 | *bla cat yqeB*^ATG→TAA^ *zeo nucB*^ATG→TAA,H47A^ | pAK91 | oAK108/oAK109 | This work |
| pAK184 | *cat* P*_spaS_*_mut_-*BlnucB*^H53A^ | pNZ8901-nucB | oAK231/oAK232 | 5 |
| pAK185 | *cat* P*_spaS_*_mut_-*BlnucB*^D93A^ | pNZ8901-nucB | oAK233/oAK234 | 5 |
| pAK186 | *cat* P*_spaS_*_mut_-*BlnucB*^D93N^ | pNZ8901-nucB | oAK235/oAK236 | 5 |
| pAK316 | *bla cat yqeB*^ATG→TAA^ *zeo* | pHM457 | oAK76/oAK77 | This work |
| pEM5 | *bla cat yqeB*^ATG→TAA^ *neo* P*_soj_*-*soj-spo0J* | pHM16 | oAK71/oAK72 | This work |
| pEM6 | *bla cat yqeB*^ATG→TAA^ *neo nucB’ spoIVCA* | pEM5 | oAK69/oAK70 | This work |
| pHM16 | *bla cat neo* P*_soj_*-*soj-spo0J* |  |  | 2 |
| pHM457 | *bla cat zeo* |  |  | 1 |
| pHM629 | *bla cat yqeB*^ATG→TAA^ *zeo nucB*^ATG→TAA,E88A^ | pAK91 | oHM584/oHM585 | This work |
| pHM630 | *bla cat yqeB*^ATG→TAA^ *zeo nucB*^ATG→TAA,N111A^ | pAK91 | oHM586/oHM587 | This work |
| pNZ8901 | *cat* P*_spaS_*_mut_ |  |  | 4 |
| pNZ8901-nucB | *cat* P*_spaS_*_mut_-*BlnucB* | pNZ8901 |  | 5 |

**Supplementary Table 3: Primer list.**

| **Product** | **Template** | **Primer #1** | **Sequence (5′→3′)** | **Primer #2** | **Sequence (5′→3′)** |
| --- | --- | --- | --- | --- | --- |
| pAK91 | Genomic DNA | oAK74 | AAGGGAAGGGGAGCTCTAAAAAAAATGGATGGCAGGC | oAK101 | AATTTAATTTCCGCGGACTACTGCACAATAAACAGC |
| pAK108 | pAK91 | oAK102 | CATCGGCCACTCAGCCCGGTCATAGCC | oAK103 | GGCTATGACCGGGCTGAGTGGCCGATG |
| pAK109 | pAK91 | oAK104 | CATCGGCCACTCATTCCGGTCATAGCCCG | oAK105 | CGGGCTATGACCGGAATGAGTGGCCGATG |
| pAK110 | pAK91 | oAK106 | TCACATATCGGACAGCAGCCCCTGCACCG | oAK107 | CGGTGCAGGGGCTGCTGTCCGATATGTGA |
| pAK111 | pAK91 | oAK108 | GCAATCGCATCCCTAATAGCACTGCCGGTTTCCGGATA | oAK109 | TATCCGGAAACCGGCAGTGCTATTAGGGATGCGATTGC |
| pAK184 | pNZ8901-*nucB* | oAK231 | TCCTGAAACCGGCGCTGCTATAAGCGACGCGATC | oAK232 | GATCGCGTCGCTTATAGCAGCGCCGGTTTCAGG |
| pAK185 | pNZ8901-*nucB* | oAK233 | GGGCTTTGACCGTGCCGAATGGCCGATGG | oAK234 | CCATCGGCCATTCGGCACGGTCAAAGCCC |
| pAK186 | pNZ8901-*nucB* | oAK235 | CAAGCCGGGCTTTGACCGTAACGAATGGCC | oAK236 | GGCCATTCGTTACGGTCAAAGCCCGGCTTG |
| pAK316 | Genomic DNA | oAK76 | AATTTAATTTGGGCCCTAATTGCAGAATCAATCTCATACTC | oAK77 | AATTTAATTTATCGATAAAGAAGAGGCTCTTTCTTG |
| pEM5 | Genomic DNA | oAK71 | AAGGAAGGGAGCTCTAATTGCAGAATCAATCTCATACTC | oAK72 | AAGGAAGGGCGGCCGCTAAAGAAGAGGCTCTTTCTTG |
| pEM6 | Genomic DNA | oAK69 | AATTTAATTTACTAGTGCCGGTTTCCGGATAACGAGAC | oAK70 | AAGGGAAGGGCTCGAGGTGATAGCAATATATGTAAGG |
| pHM629 | pAK91 | oHM584 | GGATGCTTGGCCGATGGCGGTCTGCGAG | oHM585 | GCCAAGCATCCCGGTCATAGCCCGGCTTG |
| pHM630 | pAK91 | oHM586 | CTGATGCTCGCGGCGCCGGCTCGTGGGTAG | oHM587 | CCGCGAGCATCAGAAGGCGTCACATATCGG |

**Supplementary Table 4: X-ray data collection and refinement statistics.**

| **Data collection** | S-SAD | High resolution |
| --- | --- | --- |
| Date | 27/09/12 | 14/07/12 |
| Source | I24 | I04 |
| Wavelength (Å) | 1.907 | 0.9795 |
| Space group | P4_1_2_1_2 | P4_1_2_1_2 |
| Cell dimensions |  |  |
| *a*, *b*, *c* (Å) | 59.99 59.99 66.61 | 59.96 59.96 66.60 |
| *α*, *β*, *γ* (°) | 90 | 90 |
| No. of measured reflections | 349892 (7266) | 212591 (10196) |
| No. of independent reflections | 6015 (462) | 27361 (1316) |
| Resolution (Å) | 44.58 – 2.26  (2.34 – 2.26) | 44.56 – 1.35  (1.37 – 1.35) |
| CC_1/2_ | 1.000 (0.992) | 0.991 (0.944) |
| *I*/σ*I* | 80.3 (20.3) | 16.1 (3.6) |
| Completeness (%) | 98.6 (85.4) | 100.0 (100.0) |
| Redundancy | 58.2 (15.7) | 7.8 (7.7) |
|  |  |  |
| Anomalous completeness (%) | 97.7 (76.7) |  |
| Anomalous multiplicity | 32.1 (7.9) |  |
|  |  |  |
| **Refinement** |  |  |
| *R*_work/_ *R*_free_ |  | 14.85 / 18.62 |
| No. atoms |  |  |
| Protein |  | 848 |
| Water |  | 104 |
| B-factors |  |  |
| Protein (Å^2^) |  | 18.0 |
| Water (Å^2^) |  | 26.9 |
| R.m.s deviations |  |  |
| Bond lengths (Å) |  | 0.014 |
| Bond angles (°) |  | 1.68 |

**A**

**
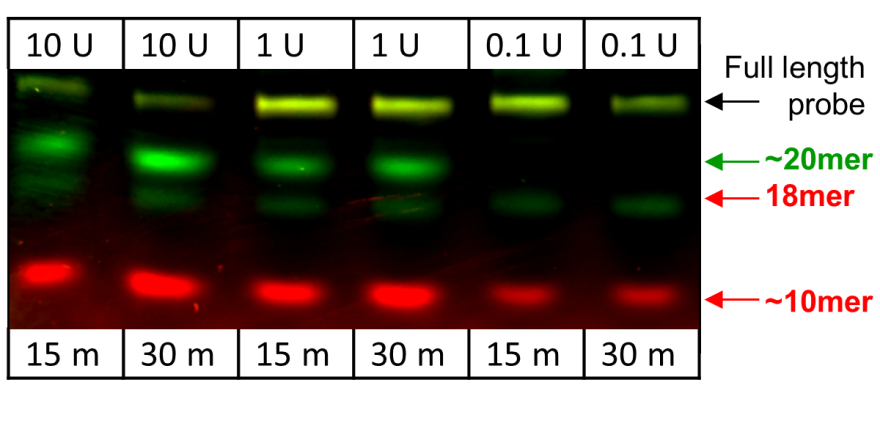
**

**B**

**
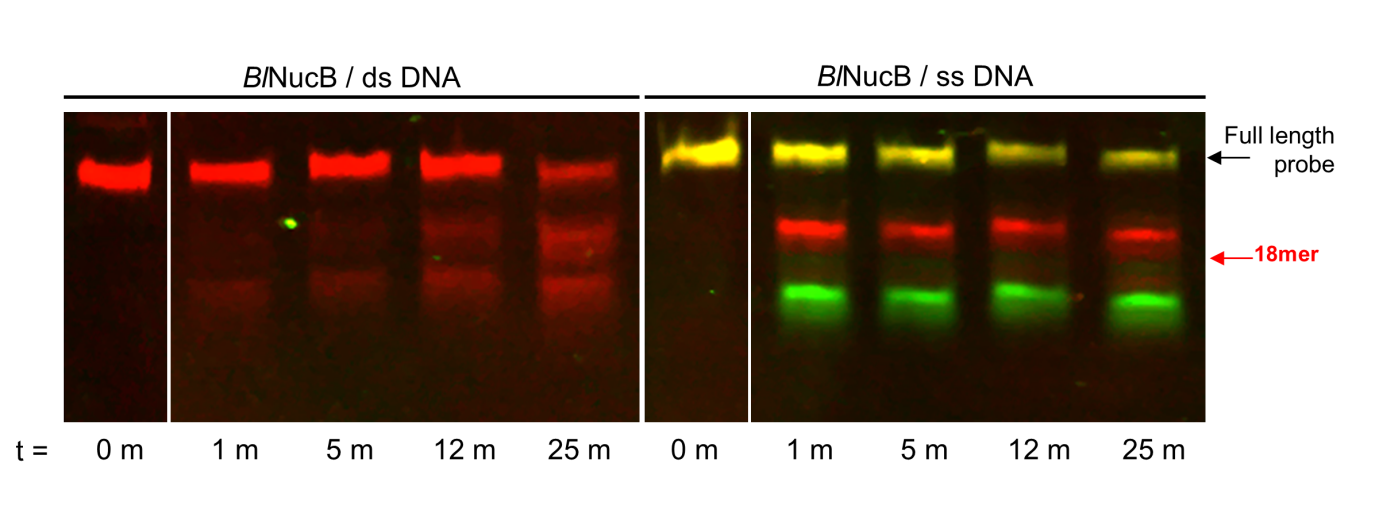
**

**Supplementary Figure 1:** **Endonuclease activity.**

(**A**) 25 nM of the dual-labelled double-stranded oligodeoxynucleotide described in Figure 2A (sequence 1 and 7) was incubated at room temperature with the non-specific Micrococcal endonuclease (NEB catalogue # M0247) for 15 and 30 minutes with the number of NEB-defined nuclease gel units added per reaction indicated above the gel. Micrococcal nuclease cleaves the oligodeoxynucleotide asymmetrically producing two clear fragments after endonucleolysis.

(**B**) 25 nM of single, FITC-labelled double-stranded oligodeoxynucleotide described in Figure 2A (sequence 2 and 7) was incubated at room temperature with *Bl*NucB at 3.33 nM for between 1 and 25 minutes (left hand panel). Note how almost all of the substrate has degraded by the end of the experiment. In the right hand panel, *Bl*NucB at 25 nM was incubated at room temperature with the single-stranded, dual-labelled oligodeoxynucleotide (sequence 1). The probe intensity continues to decline after the first time point, but not as significantly as the drop in intensity between 0 and 1 minutes, indicating that single stranded oligodeoxynucleotides are poor substrates for *Bl*NucB.


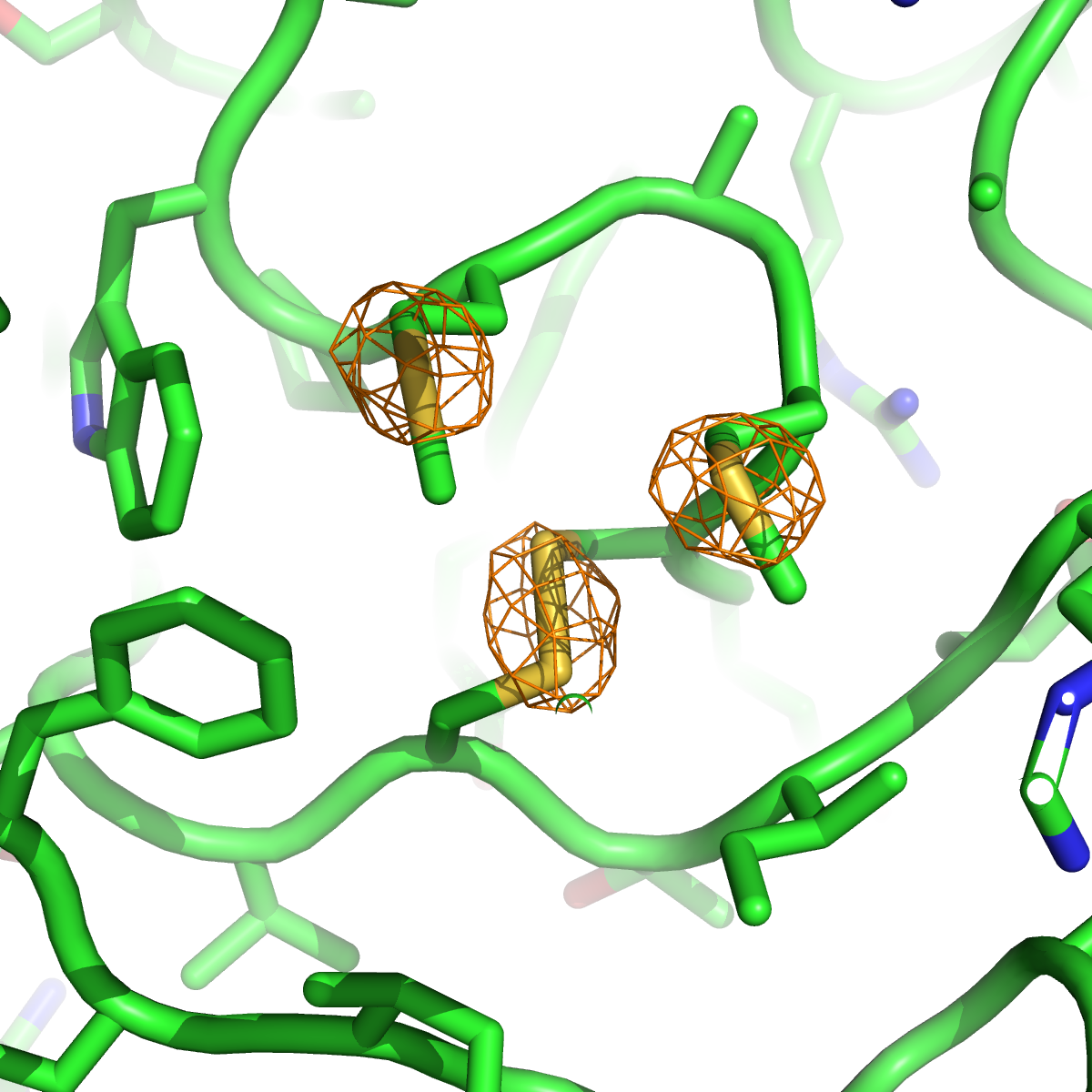
**Supplementary Figure 2: Anomalous differences in the *Bl*NucB electron density map.**

A section of the S anomalous difference map calculated with model phases and contoured at a level of 7 σ superimposed on the final, fully-refined atomic model. The only peaks in the map correspond to the four sulphur atoms from two methionines and one disulphide.


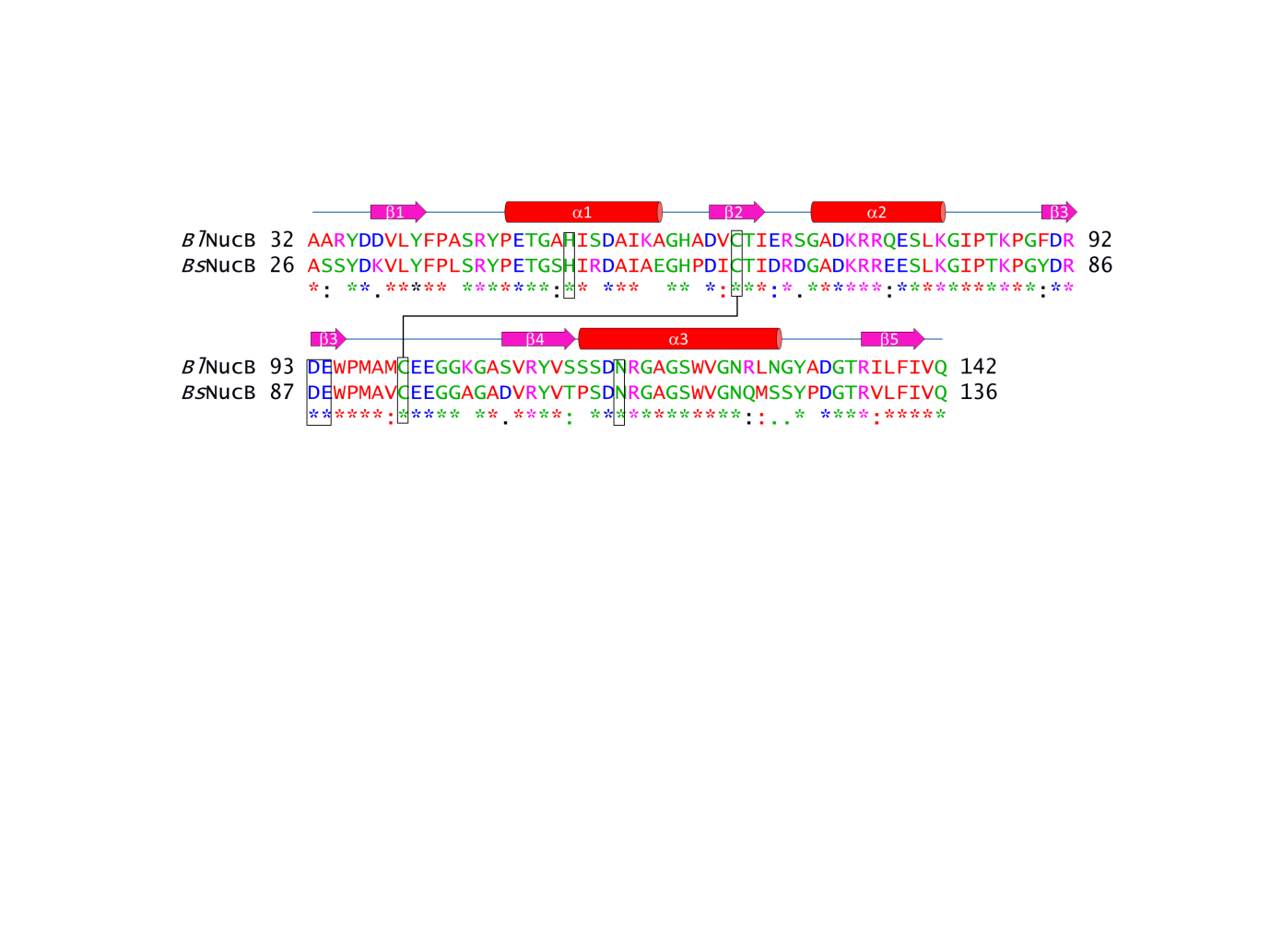


**Supplementary Figure 3**: **NucB sequence homology.**

Sequence alignment of mature *Bl*NucB vs mature *Bs*NucB, annotated with the secondary structure elements of *Bl*NucB depicted as magenta arrows (β-strands) and red cylinders (α-helices) and the residues involved in metal binding and/or catalysis are highlighted in boxes. The cysteines in the sole disulphide in *Bl*NucB are highlighted by connected boxes. Hydrophobic residues (A, F, I, L, M, P, V, W) are red, polar (C, G, H, N, Q, S, T, Y) are green, negatively charged (D, E) blue and positively charged (K, R) are cyan. Asterisks denote sequence identity, and semi-colons and dots represent strong and weak similarity, respectively.

**A**


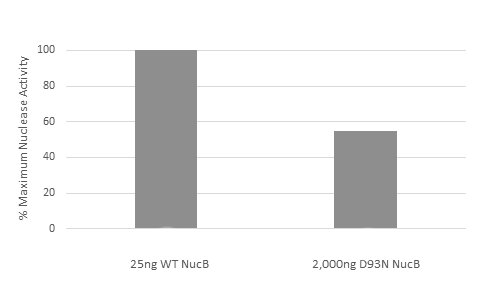


**B**


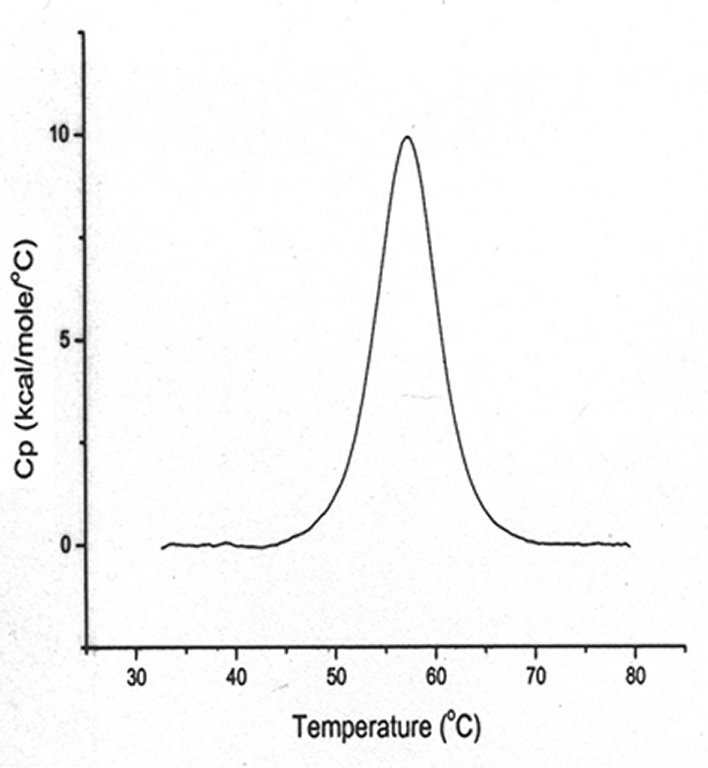

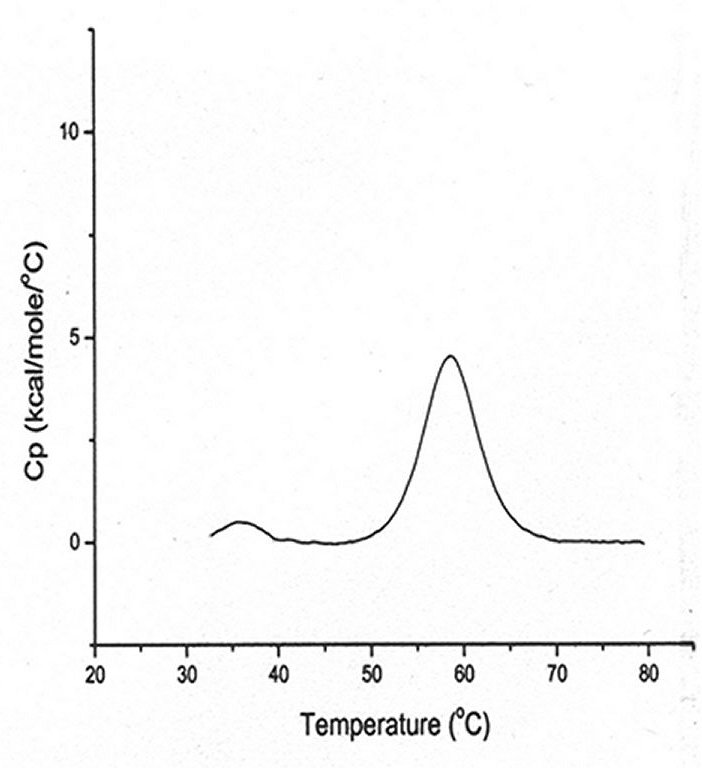


**C**


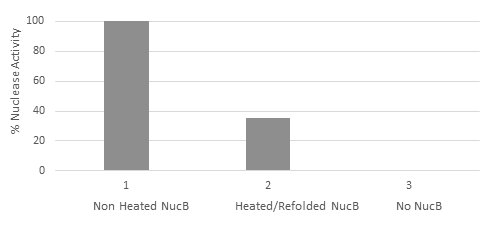


**Supplementary Figure 4: Thermostability and endonuclease activity of *Bl*NucB.**

(**A**) The relative activities of wild type and the D93N variant of *Bl*NucB are compared using high Mr calf thymus DNA as the substrate. The nuclease activity was assessed by comparing the increased absorbance of perchloric acid soluble DNA generated by the enzyme-dependent conversion of the substrate. Note that an 80-fold greater quantity of the D93N variant yielded ~55% of the activity of the wildtype enzyme and therefore the activity of the D93N mutant *Bl*NucB^D93N^ is reduced by greater than 99% compared to the WT.

(**B**) DSC reveals an average *T_m_* of the first unfolding of *Bl*NucB of 57.4 (+/-0.1) °C (left), and after spontaneous refolding on cooling, a further round of thermal unfolding yielded a *T_m_* of 58.7 (+/-0.1) °C.

(**C**) The semi-quantitative nuclease activity of unheated *Bl*NucB is normalised to 100%; when *Bl*NucB was subjected to one round of DSC-mediated thermal unfolding and spontaneous refolding, its nuclease activity was reduced by 64 %.

**A**


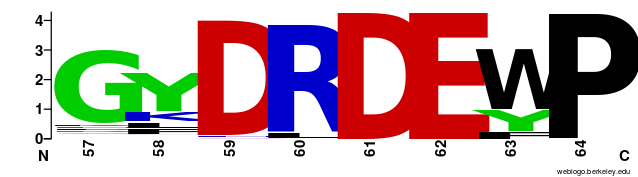


**B**


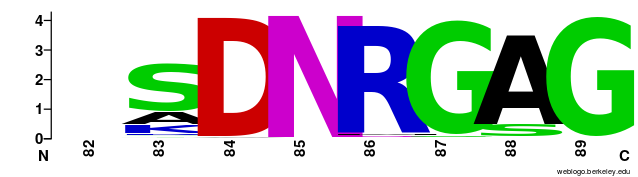


**Supplementary Figure 5: Sequence conservation of two catalytic motifs.**

Weblogos showing conservation of sequence of DRDE (**A**) and DNRG (**B**) motifs necessary for metal binding and catalysis.


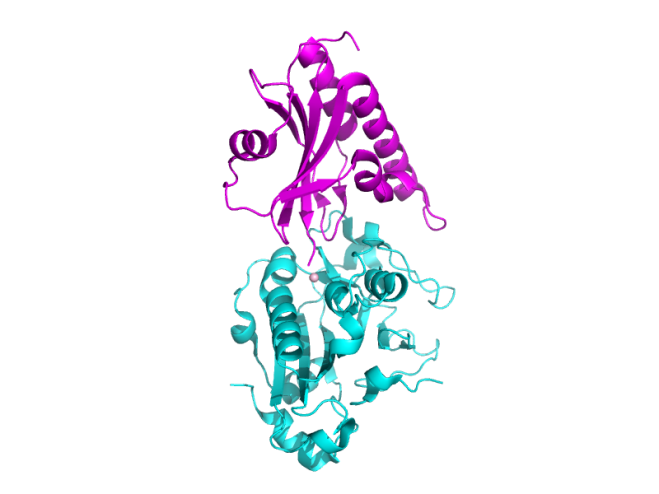


**B**

**A**


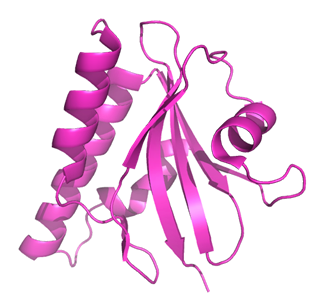

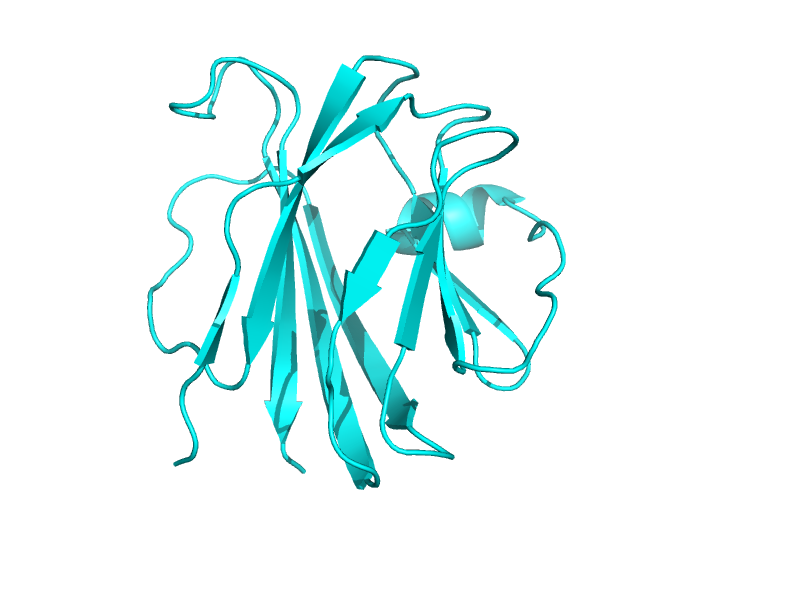


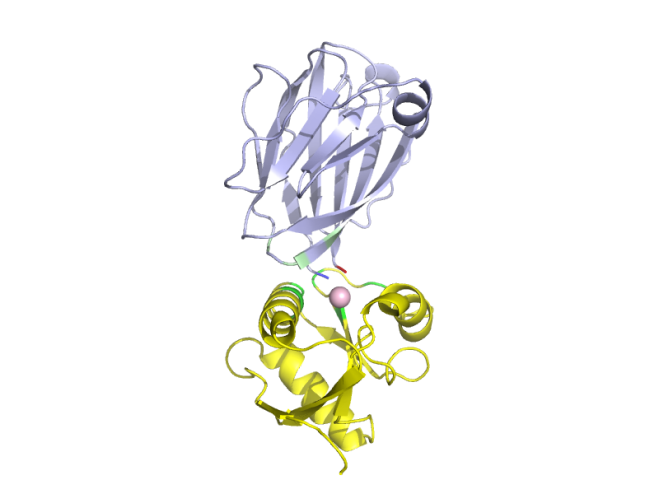


**C**

**Supplementary Figure 6: NuiA- and Nin-inhibition of nucleases.**

(**A**) Structure of *Anabaena* NucA (cyan) in complex with NuiA (pink); the bound magnesium ion is shown as a pink sphere (PDBid: 2O3B; 6).

(**B**) A comparison of the monomers of NuiA (pink; PDBid: 2O3B; 6) and Nin (cyan; PDBid: 4MQD; unpublished).

(**C**) model of Nin (blue) bound to NucB (yellow) based on the conservation of residues in likely interacting surfaces. The N- and C-terminal residues of Nin are coloured blue and red, respectively, and potential interacting residues in both proteins are coloured green. The pink sphere represents the magnesium binding site of NucB based on homology to other His-Me finger nucleases.
